# A Rat Model of Thrombosis in Common Carotid Artery Induced by Implantable Wireless Light-Emitting Diode Device

**DOI:** 10.1155/2014/724134

**Published:** 2014-06-19

**Authors:** Jih-Chao Yeh, Kuo-Lun Huang, Yung-Chin Hsiao, Yu-Han Hsu, Yun-Han Lin, Shyh-Liang Lou, Tsong-Hai Lee

**Affiliations:** ^1^Department of Biomedical Engineering, Chung-Yuan Christian University, Chung Li 32023, Taiwan; ^2^Stroke Center and Department of Neurology, Chang Gung Memorial Hospital, Linkou Medical Center and College of Medicine, Chang Gung University, No. 5, Fu-Hsing Street, Kweishan, Taoyuan 33305, Taiwan; ^3^Molecular Imaging Center, Chang Gung Memorial Hospital, Linkou 33305, Taiwan

## Abstract

This work has developed a novel approach to form common carotid artery (CCA) thrombus in rats with a wireless implantable light-emitting diode (LED) device. The device mainly consists of an external controller and an internal LED assembly. The controller was responsible for wirelessly transmitting electrical power. The internal LED assembly served as an implant to receive the power and irradiate light on CCA. The thrombus formation was identified with animal sonography, 7T magnetic resonance imaging, and histopathologic examination. The present study showed that a LED assembly implanted on the outer surface of CCA could induce acute occlusion with single irradiation with 6 mW/cm^2^ LED for 4 h. If intermittent irradiation with 4.3–4.5 mW/cm^2^ LED for 2 h was shut off for 30 min, then irradiation for another 2 h was applied; the thrombus was observed to grow gradually and was totally occluded at 7 days. Compared with the contralateral CCA without LED irradiation, the arterial endothelium in the LED-irradiated artery was discontinued. Our study has shown that, by adjusting the duration of irradiation and the power intensity of LED, it is possible to produce acute occlusion and progressive thrombosis, which can be used as an animal model for antithrombotic drug development.

## 1. Introduction

With the increasing population of elderly people, stroke has become a major health issue and the leading cause of death worldwide. Thrombosis is a diffuse pathologic process that starts with endothelial dysfunction and clinically manifests as coronary artery disease, cerebrovascular disease, and transient ischemic attack [1]. Considering the critical role of thrombosis formation in many vascular diseases, several animal models have been developed to study the underlying mechanism and therapeutic options, such as mechanical [2] or electrical trauma [3, 4], vessel ligation [5], ferric chloride-induced injury [6–9], and photochemical injury [10–15]. Recently, arterial thrombosis induced by photochemical injury has become a widely used animal model. The photosensitizing dye, rose bengal, which is used in photochemical injury is administrated intravenously into the circulation and then exposed to green light at 543 nm to generate singlet oxygen radicals and cause direct oxidative injury to endothelial cells. The photochemical injury model is likely to be similar to the mechanism underlying human arterial thrombosis caused by reactive oxygen species.

Using laser-induced photochemical injury for thrombosis generation, the location and precise time can be controlled; however, the power of laser irradiation is too strong to cause severe endothelial injury [15, 16] and rapid accumulation of platelets at the vessel wall [12] leading to immediate thrombotic occlusion. These findings suggest that a lower power light source and longer irradiation time should be advantageous for this application of thrombosis formation. Thus, we selected light-emitting diode (LED), because the light power is less and control is easier. In addition, the advantages of LED include inexpensiveness, low energy consumption, long life time, small size, and fast switching [17].

Considering the issue of long irradiation time, a wireless implantable LED device has been developed. No internal battery is needed in this device; thus, the concerns of limited lift time, large volume, and possibility of leakage from the internal battery [18] are eliminated. In addition, the device is also capable of wirelessly transferring electric power; thus, the LED implantation avoids transcutaneous wiring.

This work describes the* in vivo* wireless implantable LED device and establishes a new rat model of thrombosis. The temporal changes in thrombus formation were examined by using animal sonography, 7T magnetic resonance imaging, and immunohistochemical examination.

## 2. Materials and Methods

### 2.1. *In Vivo* LED-Induced Thrombosis Model

#### 2.1.1. Wireless Implantable LED Device

**Figure 1:**
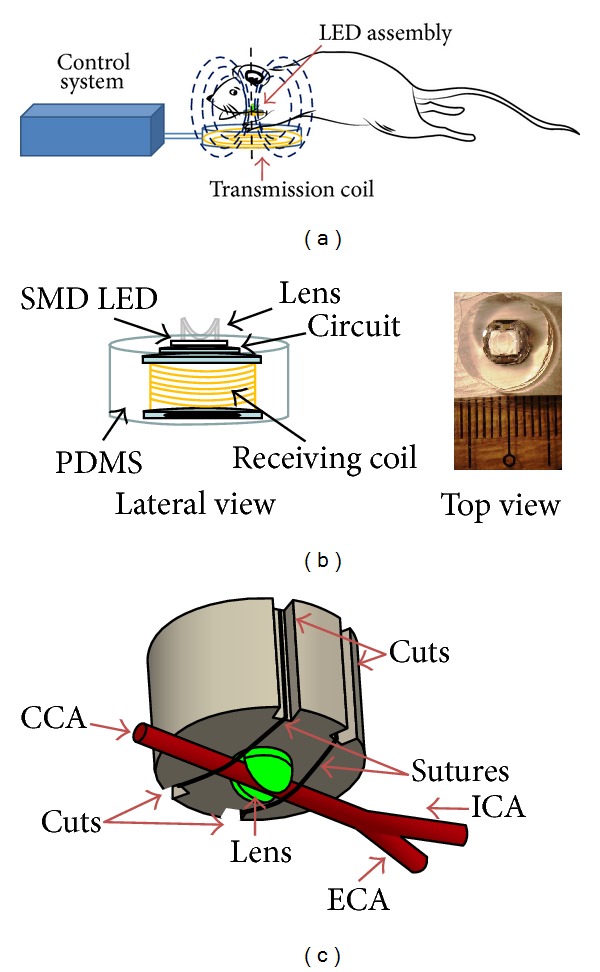
Schematic description of the LED irradiation apparatus. (a) The irradiation apparatus is composed of control system, transmission coil, and LED assembly. (b) The LED assembly consists of SMD LED, receiving coil, and associated circuit, which are hermetically packaged with PDMS. (c) The schematic diagram of implanting the LED assembly around carotid artery. CCA: common carotid artery; ECA: external carotid artery; ICA: internal carotid artery.

The homemade wireless implantable LED device consists of two parts (an external power controller for wirelessly transmitting electric power and an internal LED assembly to be implanted in an artery for lighting). The controller mainly includes a Class-E amplifier and a resonant transmitting coil (90 mm in diameter). The LED assembly consists of a resonant receiving coil (7 mm in diameter) and a surface-mount device type LED (2.5 × 3 × 3 mm [*W* × *L* × *H*]; Figure 1(a)). The external controller wirelessly transports electric power to the internal LED assembly via the electromagnetic wave (1 MHz) between the two coils (Figure 1(b)). Note that in Figure 1(c) the LED and the receiving coil are hermetically packaged with polydimethylsiloxane (PDMS) [19]. The assembly is approximately 10 mm in diameter and 7 mm in height. Effort was made on the LED assembly to fix a common carotid artery. An open ditch of approximately 2 mm in width and depth was made on the LED lens, and 4 cuts (2 × 2) were on the PDMS. The open ditch serves as a bed for the common carotid artery and the four cuts are prepared for suturing.

The light power of LED was measured by a Nova handheld laser power meter (Ophir Optronics Solutions Ltd., Israel). Note that the light power (*P*_L_) emitted by the LED depends on the electrical power received by the receiving coil (*P*_R_). *P*_R_ is in general inversely proportional to the distance between the transmission coil and the receiving coil (*d*_TR_) and is proportional to the electrical power from the transmission coil (*P*_T_). *P*_T_ in our device is adjustable. Prior to each experiment, *d*_TR_ was set at 3 mm and then a knob in the external control system was adjusted for regulating the power of LED till a predetermined *P*_L_ was met such as 6 mW/cm^2^ for the acute occlusion and 4.3 ~ 4.5 mW/cm^2^ for progressive thrombosis. Setting *d*_TR_ to 3 mm is based on the measurement of the thickness of skin and tissues surrounding the LED assembly at the suturing area.

#### 2.1.2. Animals

All surgical procedures and postoperative care were performed following the Guide for the Care and Use of Laboratory Animals of Chang Gung Memorial Hospital. Thirty-five male spontaneously hypertensive rats (SHRs) weighing 300–350 g and in the age of 14–18 weeks were purchased from the National Laboratory Animal Center for this study. All of the rats were provided with a standard diet and tap water* ad libitum*.

#### 2.1.3. Surgical Procedure and Photochemically Mediated Thrombosis

Twenty-four hours prior to surgery, the systolic blood pressure (SBP) and heart rate were measured using the indirect tail cuff method with a noninvasive blood pressure meter (BP-98A) in awake rats (Softron Co. Ltd., Tokyo, Japan). The LED used in this work was sterilized with 75% alcohol and ultraviolet radiation overnight prior to implantation.

Before anesthetizing the animals, the rats were carefully handled to minimize the stress, quickly anesthetized with 3% isoflurane in a mixture of 30% oxygen and 70% nitrogen, and then maintained at 1%–1.5% isoflurane during surgery and LED induction. After the administration of anesthesia, the rats were placed in the supine position. A 1 cm incision was made in the left inguinal area and the femoral vein was carefully dissected. A polyethylene tube (PE-10) (Becton, Dickinson and Company, Franklin Lakes, New Jersey, USA) to be used for rose bengal injection was inserted in the femoral vein via the dissected incision and secured using 3-0 nylon suture. Another procedure with a transverse incision in the inferior aspect of the sternal manubrium was made. Blunt dissection of the left common carotid artery (CCA) was performed. The CCA was laid in the ditch on the LED lens and then the CCA was fixed on the LED assembly by using suture to wrap them along the four cuts (Figure 1(c)). After the implantation, the surgical wound was sutured and rats were placed in prone position on the transmission coil of the external controller.

After isoflurane was decreased to 1%, rose bengal (60 mg/kg; Sigma-Aldrich, St. Louis, MO, USA) was administrated intravenously as the photosensitizing dye. By powering on the external controller, the CCA was irradiated by the implanted LED (540 nm). After irradiation, the implant was removed and the wound was sutured using 3-0 nylon. The rats were allowed to return to their cages, and food and water were provided* ad libitum*.

#### 2.1.4. Animal Groups

**Figure 2:**
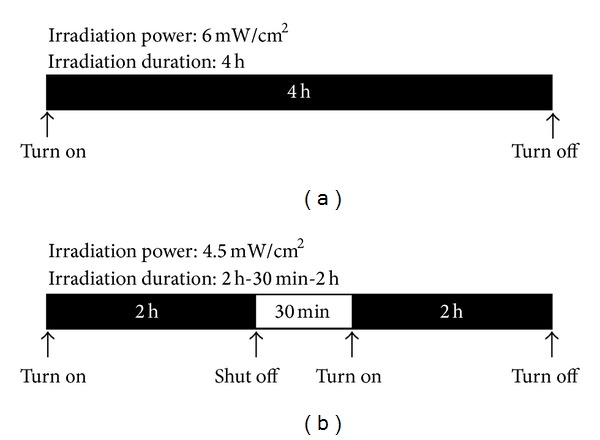
The experimental protocol of the (a) acute thrombosis and (b) progressive thrombosis models.

Four groups of SHRs were examined for comparison. The SHRs in group 1 (Figure 2(a) (acute thrombosis model)) were subjected to single irradiation to form acute occlusion. The protocol of single irradiation with LED was set for 4 h duration and 4–6 mW/cm^2^ power to investigate the effect of LED on thrombosis generation. After repeated examinations, it was determined that 6 mW/cm^2^ LED irradiation for 4 h yielded acute occlusion of the CCA.

To establish a progressive thrombosis model, the SHRs in group 2 were subjected to intermittent irradiation. As shown in Figure 2(b), 4.5 mW/cm^2^ of LED was used in the progressive thrombosis model. The irradiation was induced for 2 h first, shut off for 30 min, and then induced again for another 2 h. The SHRs in group 3 were subjected to LED irradiation alone for 4 h without the intravenous administration of rose bengal on the left CCA. The right CCAs in the acute thrombosis model, progressive thrombosis model, and LED irradiation alone were used as controls and assigned to only receive intravenous administration of rose bengal to investigate the effect of chemical irritation on the vessel wall.

### 2.2. Serial* In Vivo* Detection of Thrombosis Progression by Ultrasonography

Thrombosis progression in the CCA was examined before surgery, immediately after LED irradiation, and at 3 and 7 days after irradiation. Rats were quickly anaesthetized with 3% isoflurane in a mixture of 30% oxygen and 70% nitrogen and maintained at 1.5% isoflurane during ultrasonography. High resolution B-mode and pulse-wave Doppler were performed using a high resolution imaging system (Vevo 2100) (VisualSonics, Toronto, Ontario, Canada) equipped with a 40 MHz transducer (MS 550D) from VisualSonics. The transverse scans of the CCA were located; then the probe was rotated 90° to obtain longitudinal images. The intima-media thickness and lumen diameter of the CCA were examined from B-mode image. The change in the velocity of blood flow was measured by pulse-wave Doppler. The stenotic ratio was calculated by [(the lumen diameter in CCA− the diameter in the narrowest site caused by thrombus)/the lumen diameter in CCA] × 100%.

### 2.3. Serial* In Vivo* Imaging of Thrombotic Plaque by Magnetic Resonance (MA) Angiography

**Figure 3:**
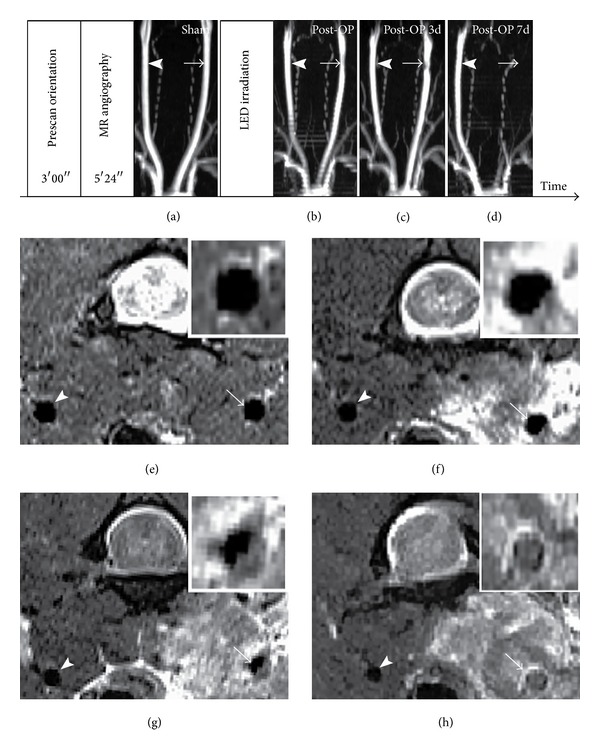
The experimental scheme of typical MR imaging and the axial T2-weighted images of the time-dependent changes of thrombus formation after intermittent 4.5 mW/cm^2^ LED irradiation for 2 h first, shut off for 30 min and for another 2 h. The MR angiogram indicates bilateral common carotid arteries before LED irradiation (a, sham) and immediately (b, immediately postoperative), 3 days (c, postoperative 3 d), and 7 days (d, postoperative 7 d) after LED irradiation. As shown in the figure, the size of the thrombus is increased by time and there is no artery signal (arrow) at 7 days (d). The axial T2-weighted image demonstrates the temporal changes of thrombus formation before LED irradiation (e, sham) and immediately (f, postoperative), 3 days (g, postoperative 3 d), and 7 days (h, postoperative 7 d) after LED irradiation. The arrows indicate the thrombus in the LED-irradiated left common carotid artery (CCA), and the arrowheads indicate the non-LED-irradiated right CCA. For comparison, please refer to the ultrasound images of CCA in Figure 5 and the histologic and immunohistochemical staining of arterial thrombosis in Figure 6. All these three (Figures 3, 5, and 6) demonstrate the change at CCA after intermittent 4.5 mW/cm^2^ LED irradiation.

Serial* in vivo* MRA examinations [20] were performed on rat CCAs and the typical MRI experimental scheme of the time sequence is shown in Figure 3. Images were acquired using a 7T MRI system (ClinScan 70/30 USR) (Bruker, Rheinstetten, Germany) with a linear body coil. The rats were anaesthetized with 1.5% isoflurane and connected to a respiratory rate monitor. The flow of anesthetic gas was constantly regulated to maintain the breathing rate at approximately 40/min. MR angiography of the CCA was performed using the time-of-flight (TOF) method without a contrast agent. Transverse slices were acquired with a fast, low-angle shot (FLASH) using the following parameters: TR = 22 ms, TE = 4.87 ms, pulse angle = 90 degrees, field of view (FOV) = 55 × 42 mm^2^, and matrix size = 58 × 256. T2-weighted images were acquired using a turbo spin-echo sequence as follows: TR = 2900 ms, TE = 37 ms, turbo factor = 7, FOV = 45 × 45 mm^2^, slice thickness = 1 mm, and matrix size = 240 × 320. The imaged area covered the CCA, the carotid bifurcation, the internal carotid artery, and the external carotid artery. Contiguous cross-sectional images were obtained perpendicular to the long axis of the neck. In each animal, the contralateral right CCA was used as a negative control.

### 2.4. Histologic and Immunohistochemical Studies

At the end of each experiment, the animals were anesthetized and sacrificed. Both CCAs were removed and washed in phosphate buffer solution, dehydrated on dry ice, and stored at −80°C for 24 h. Before sectioning, all CCAs were embedded in Optimal Cutting Temperature (Tissue Tek, 4583) (Sakura Finetek USA, Inc., Torrance, California, USA) and stored at −80°C for another 12 h. Cross-sections of the CCA (10 *μ*m thickness) were consecutively cut on a microtome-cryostat. Then, these sections were subjected to hematoxylin and eosin (H&E) staining for evaluation of thrombus formation. For immunohistochemical staining, the slides were treated with cold acetone for 10 min and then with methanol with 0.3% H_2_O_2_ for 10 min. After blocking endogenous peroxidase using a peroxidase blocking reagent (DAKO, Carpinteria, CA, USA), the primary antibody (anti-PECAM-1 [anti-CD31]) (AbD Serotec, Dusseldorf, Germany) was applied at a dilution of 1 : 500 overnight at 4°C, followed by a universal immunoperoxidase polymer (Histofine) (Cosmo Bio, Carlsbad, California, USA) for 1 h at room temperature. Development of color was achieved by exposure for 2 min to the DAB chromogen system (DAKO). Endothelium cells were considered positive for PECAM-1 in the presence of intense brown staining. The contralateral non-LED-irradiated CCA was used as a control.

## 3. Results

### 3.1. Acute Thrombotic Occlusion Model Induced by Single LED Irradiation

**Figure 4:**
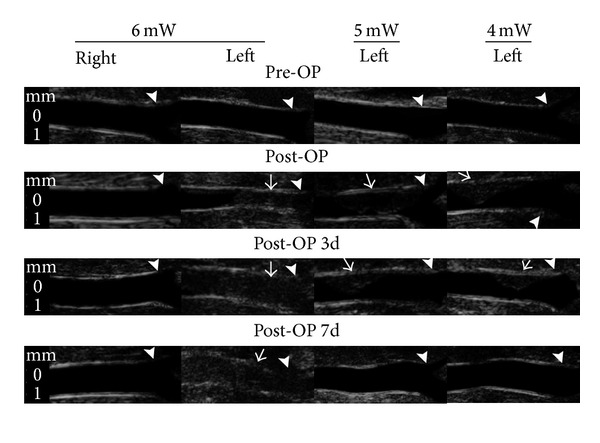
Ultrasound images of common carotid artery (CCA) after single LED irradiation with 6, 5, and 4 mW/cm^2^ for 4 h. The CCA is occluded immediately after 6 mW/cm^2^ LED irradiation. However, CCA is severely stenotic immediately after 4 and 5 mW/cm^2^ irradiation but is found to be recanalized at 7 days after irradiation. Pre-OP: before LED irradiation; post-OP: immediately after LED irradiation; post-OP 3 d: 3 days after LED irradiation; post-OP 7 d: 7 days after LED irradiation. The non-LED-irradiated right CCA is used as a control. White small arrowheads indicate the CCA bifurcation. White arrows demonstrate the formation of thrombus in the CCA.

Fifteen SHRs were used to establish the acute thrombosis model. The SBP and heart rate values prior to surgery were 221 ± 20.5 mmHg and 433 ± 41 beats per minute, respectively. To induce acute thrombotic occlusion, the LED was implanted onto the left CCA and irradiated once for 4 h at various powers (4, 5, and 6 mW/cm^2^) with intravascular administration of 60 mg/kg of rose bengal. Upon irradiation, the thrombus was observed at the irradiated site. The carotid ultrasound was scanned for confirmation, and as shown in Figure 4 the left CCA in the 6 mW/cm^2^ group was totally occluded after irradiation and the blood velocity was 0 mm/sec as measured by pulsed-wave Doppler mode.

In the 4 and 5 mW/cm^2^ groups, the thrombus was also formed within the region irradiated by the LED; however, 3 days after irradiation, the thrombus was gradually diminished and disappeared at 7 days. In addition, the contralateral CCA (right) received intravenous administration of rose bengal alone without LED irradiation, and no thrombosis was visualized on the ultrasound images. Therefore, thrombus formation was induced by LED irradiation with rose bengal intravenous administration.

### 3.2. Progressive Thrombosis Model Induced by Intermittent LED Irradiation

Fifteen SHRs were used to develop the progressive thrombosis model. The SBP and heart rate values prior to surgery were 223 ± 7.5 mmHg and 395 ± 46 beats per minute, respectively. To establish the progressive thrombotic occlusion model, 4-5 mW/cm^2^ of LED was examined with first irradiation for 2 h, shut off for 30 min, and a second irradiation for another 2 h. When the power of LED was >4.5 mW/cm^2^, the thrombus was formed at the LED irradiation site, and then the CCA was found totally occluded 1 day after irradiation. Using 4.3–4.5 mW/cm^2^ of the LED, the thrombus formation was gradually enlarged at the region irradiated by the LED. In contrast, if the LED power was <4.3 mW/cm^2^, the thrombus appeared only transiently and disappeared within 1 day after irradiation. In addition, LED irradiation only or rose bengal injection only caused vessel wall injury but no thrombosis formation. Based on the above studies, it was noted that intermittent irradiation by 4.3–4.5 mW/cm^2^ LED combined with intravenous administration of 60 mg/kg of rose bengal was the most suitable condition for the establishment of progressive thrombosis model. The carotid ultrasound, MR angiography, and histologic and immunohistochemical studies were used to further confirm thrombus generation in the progressive thrombosis model.

#### 3.2.1. Ultrasound Imaging of the CCA with Intermittent Irradiation

**Figure 5:**
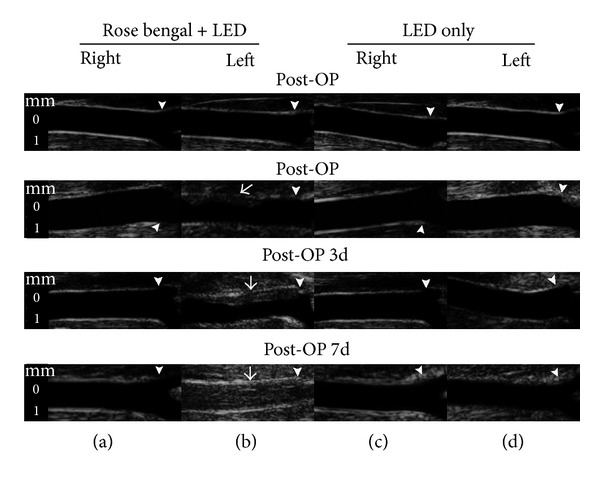
Ultrasound images of common carotid artery (CCA) after intermittent LED irradiation Using 4.5 mW/cm^2^ LED irradiated for 2 h first, shut off for 30 min, then for another 2 h. In the left CCA with rose bengal injection and intermittent LED irradiation (b), there is progressive stenosis after irradiation to total occlusion at 7 days. However, in the right CCA with rose bengal injection only (a), left CCA with LED irradiation only (d), and right CCA with neither rose bengal injection nor intermittent LED irradiation (c), there is no remarkable carotid stenosis. Pre-OP: before LED irradiation; post-OP: immediately after LED irradiation; post-OP 3 d: 3 days after LED irradiation; post-OP 7 d: 7 days after LED irradiation. The non-LED-irradiated right CCA is used as control (a and c). Rose bengal + LED indicates that after intravenous administration of 60 mg/kg rose bengal CCA is irradiated intermittently with 4.5 mW/cm^2^ LED. LED only indicates that the CCA was irradiated by LED without rose bengal administration. Small white arrowheads indicate the CCA bifurcation. White arrows demonstrate the formation of thrombus in the CCA.

Figure 5 is the ultrasound images of thrombus formation in the left CCA after intermittent 4.5 mW/cm^2^ LED irradiation. After LED irradiation, the thrombus was formed at the region irradiated by LED and the lumen was narrowed to 30% (the narrowest site). The thrombus gradually enlarged in size at 3 days and the lumen was narrowed to 45%. Finally, the artery was totally occluded 7 days after irradiation and the blood flow velocity measured by pulse-wave Doppler was 0 mm/sec.

#### 3.2.2. MR Imaging of the CCA

Thrombus formation in the left CCA after intermittent 4.5 mW/cm^2^ LED irradiation was observed by MR angiography and coronal sequential MR images. Before LED irradiation, the vessel lumen in MR angiography and T2-weighted images was not significantly different between the right and left CCAs. After LED irradiation, the thrombus appeared on the vessel wall of the left CCA (Figure 3(f)). Because the blood flow of the left CCA was disrupted by thrombus, MR angiography of the left CCA was not as bright as the right CCA (Figure 3(b)). Three days after irradiation, as shown in Figures 3(c) and 3(g), the thrombus was enlarged and significantly disrupted blood flow. Seven days after irradiation, the left CCA was totally occluded (Figure 3(h)) and caused signal disappearance of the left CCA on MR angiography (Figure 3(d)).

#### 3.2.3. Histopathologic Findings of Arterial Thrombosis

**Figure 6:**
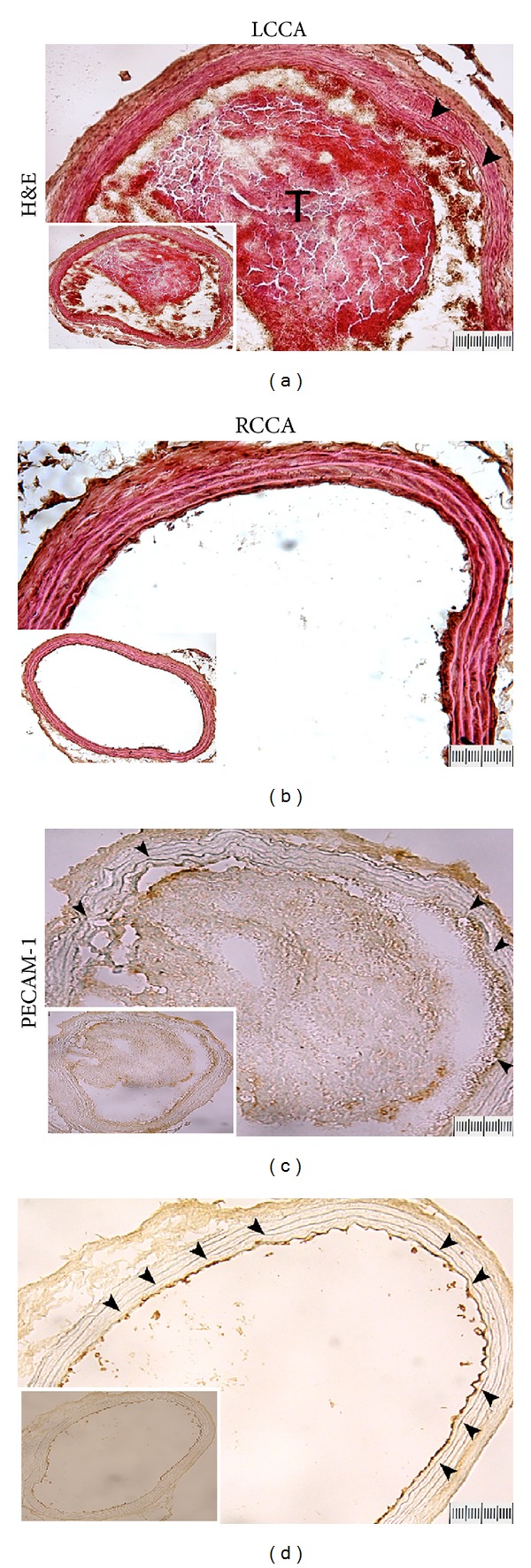
Histologic and immunohistochemical staining of arterial thrombosis 3 days after intermittent 4.5 mW/cm^2^ LED irradiation for 2 h first, shut off for 30 min, then for another 2 h. Microphotographs of hematoxylin-eosin (a and b) and PECAM-1 (c and d) staining at the left common carotid artery (CCA) with LED irradiation and right CCA without LED irradiation. Thrombus formation can be seen in the lumen of the LED-irradiated vessel (a and c). The endothelium of the LED-irradiated vessel is injured and there is platelet adhesion (a, arrowheads). PECAM-1 expression in injured and noninjured vessels is shown in c and d. Except for the platelet adhesion, the endothelial cells are injured and discontinued (c, arrowheads). However, the endothelial cells in the CCA without LED irradiation are intact (d). T: thrombus. Original magnification 200x. Bar = 200 *μ*m.

Thrombus composition and endothelial damage after intermittent 4.5 mW/cm^2^ LED irradiation were studied by staining with H&E and anti-PECAM-1. Histologic examination of the CCAs after intermittent LED irradiation for 3 days (Figure 6(a)) showed that the CCA with LED irradiation had formation of thrombus packed with red blood cells, leucocytes, and platelets, whereas no thrombus formation was observed in the contralateral (no LED irradiation) CCA (Figure 6(b)). The endothelium of the LED-irradiated vessel was injured and platelet adhesion occurred. In contrast, the contralateral noninjured CCA wall remained smooth and thin. PECAM-1 expression in injured and noninjured vessels is shown in Figures 6(c) and 6(d). After intermittent LED irradiation, except for the platelet adhesion, the endothelial cells were also injured and discontinued; however, the endothelial cells in the CCA without LED irradiation were intact.

## 4. Discussion

Thrombosis in the major arteries supplying the heart or brain is a threatening event leading to morbidity and mortality. Several animal thrombosis models have been developed to explore the pathophysiology of thrombosis to prevent vascular diseases, such as myocardial infarction and stroke; however, most thrombosis models have been established by using chemical damage or photochemical injury. The chemical damage model using ferric chloride causes severe endothelial damage and vessel occlusion. This ferric chloride method produces transmural cell necrosis and interruption of the vascular endothelium integrity. Of note, this model is simple and no specialized equipment is needed to investigate the mechanism underlying thrombosis. The occlusion time depends on the concentration of ferric chloride (10%–50%) and the size of the filter paper [7–9]. However, ferric chloride usually causes an acute thrombosis model on small vessels in mice and the precise mechanism underlying the thrombosis is still not well defined.

Reactive oxygen species are thought to be important contributors to endothelial damage [21] and platelet accumulation [22] leading to atherosclerosis and thrombosis. Photochemical reactions may produce singlet oxygen, promote damage to the vascular endothelium by reactive oxygen species, and then initiate thrombus generation, which is more similar to the mechanism underlying human arterial thrombosis. Therefore, some researchers have tried to use a photothrombotic technique to induce small vessel occlusion by the combination of a photosensitizing dye and an appropriate light source. Argon [11], argon-dye laser beam [12], and krypton laser system [23] are the conventional light sources used in the photothrombotic model. However, these devices have some disadvantages mostly due to technical difficulties, large size of the equipment, heat production, induction of tissue damage, and high expense. In addition, using argon or argon-dye laser at 540 nm as the light source reduces the absorbed efficiency of rose bengal and needs a much longer irradiation time to cause vessel occlusion [12, 24]. The LED used in our model has several advantages including low energy consumption, long lift time, small size of equipment, fast switching, and high durability and safety. Therefore, the LED can replace the laser as an ideal light source to induce arterial thrombosis by photochemical injury.

To miniaturize the size and transfer energy to the* in vivo* implantable LED light source, a wireless energy transfer system based on the near field transmission method [25–28] was designed in this implantable LED device. Near field transmission involves inductive and magnetic resonance coupling. Recently, it has been found that two coupling coils with the same intrinsic resonant frequency exchange energy are more efficient than two nonresonant coils [29]. The power transfer efficiency is affected by the relative location between the transmitter and receiver coils [30]. In our* in vivo *LED induction thrombosis model, the resonant frequency of the transmitter and receiver coils was 1 kHz. The power transfer efficiency is maintained at 90% at a distance within 6 mm between the transmitter and receiver coils. In addition, the magnetic field interacts weakly with tissues or organisms, making near field transmission a relatively safe method for power transfer. Therefore, the implantable LED device is a stable and safe apparatus for the* in vivo* LED-induced thrombosis model.

The present induced thrombosis model is based on a photochemical reaction to produce thrombotic occlusion by intravenous injection of rose bengal and irradiation with green light from an implantable* in vivo* LED induction light source. Rose bengal is used as a type II photosensitive dye [31] and accumulates in the lipid bilayer of the endothelial cells, which can be excited in the bloodstream, induce reactive oxygen species, and cause peroxidative damage of the endothelial membrane. The damaged endothelial cells may result in platelet adhesion, erythrocyte aggregation, and fibrin mesh mixed with platelets, which occurs mostly at the interface with the injured vessel [32].

Using our implantable LED device can establish acute and progressive thrombosis models by adjusting the duration of irradiation and power intensity of the LED. The acute thrombosis model could be obtained using a 6 mW/cm^2^ LED and single irradiation for 4 h. The LED irradiation needs a longer duration because the power of the LED is smaller than the laser (15 W/cm^2^) [33]. Furthermore, the progressive thrombosis model could be acquired using a 4.3–4.5 mW/cm^2^ LED with irradiation for 2 h first, shut off for 30 min, and irradiation for another 2 h. Because the power of the LED is smaller than laser, the heat produced by LED irradiation is negligible. Therefore, the tissue damage is less as compared with the laser induction model. The rats used in the acute or progressive thrombosis model can survive during the experiment period before being sacrificed.

The present LED induction method has several advantages. First, the LED enables focal irradiation with the diameter of an irradiation area as small as 2-3 mm, so the illuminated area is confined to the CCA. In addition, a 1-2 mm diameter ditch on the lens is used in this LED irradiation system, and the damage is minimized on the targeted vessel. Second, this LED system is composed of an* in vitro* energy transmitting control system, resonant transmitter coil, and an* in vivo* LED induction light source. The power intensity of the LED can be regulated through the* in vitro* transmitting control system. Based on the inductive coupling principle, a wireless energy transfer system is designed and implemented for the power supply to the LED light source. Third, the* in vivo* LED induction light source derived from the* in vitro* transmitter control system has good stability and produces higher light levels with low radiant heat than high-pressure lamps or laser to avoid hyperthermic injury. Fourth, the whole* in vivo* LED induction light source is packaged with polydimethylsiloxane (PDMS), which secures a better seal and biocompatibility.

In conclusion, our study provides an* in vivo* wireless implantable LED device which can be used to induce thrombus generation in the CCA. Acute and progressive thrombosis models can be obtained by regulating the duration of irradiation and power intensity of the LED. This LED irradiation system offers a simple, reproducible, and inexpensive method for evaluation of the potential therapeutic agents against artery thrombosis.
